# Complete genome sequence of *Cellulosilyticum lentocellum* HNost1007, a cellulolytic strain isolated from ostrich feces

**DOI:** 10.1128/mra.00648-26

**Published:** 2026-10-21

**Authors:** Yumiko Ogawa, Miku Fujita, Tomomi Ban-Tokuda, Hiroki Matsui

**Affiliations:** 1 Graduate School of Bioresources, Mie University 12946, Tsu, Mie, Japan; University of Maryland School of Medicine, Baltimore, Maryland, USA

**Keywords:** anaerobic, cellulolytic, ostrich feces

## Abstract

The complete genome sequence is described from *Cellulosilyticum lentocellum* HNost1007, cellulolytic bacteria isolated from ostrich feces. The genome contains a 4,639,609 bp chromosome, 34.4% GC content, 4,262 coding sequences, 25 ribosomal RNAs, and 103 transfer RNAs.

## ANNOUNCEMENT

The ostrich (*Struthio camelus*) possesses a notably enlarged hindgut compared to other avian species (1). This species is recognized as an herbivorous bird, capable of digesting plant cell walls in its hindgut. Our initial study characterized the microbiota composition in the ceca of adult ostriches using a 16S ribosomal RNA (rRNA) gene clone library (2), revealing a highly complex microbial community. Efforts to isolate fibrolytic microorganisms had been lacking for an extended period until *Fibrobacter intestinalis*, identified through molecular techniques, was isolated from ostrich feces (3). This was the first *Fibrobacter* isolate from a non-mammalian species, capable of utilizing crystalline cellulose as its sole carbon source (3). Recently, we isolated *Cellulosilyticum* (formerly *Clostridium*) *lentocellum* HNost1007 from ostrich feces, and in this study, we present its complete genome sequence. *C. lentocellum*, a cellulolytic bacterium, was first isolated from river sediment containing paper mill waste (4). *C. lentocellum* has also been isolated from the feces of Blue Budgerigars (5), cattle (6), and giant pandas (7). A freshly voided fecal sample was non-invasively collected from an adult ostrich at Hisai Norin High School (Mie Prefecture, Japan: 34.692860, 136.477457). The sample was placed in a plastic bag and maintained at 39°C during transport to our laboratory, arriving within 30 min. The isolation procedure for *C. lentocellum* followed the techniques as described previously (8, 9). Briefly, a slurry of the feces was inoculated into enrichment medium that contains 12 × 12 mm Whatman No. 1 filter paper and incubated at 39°C. The partially decayed filter paper was transferred to anaerobic dilution buffer and cooled on ice for 30 min and then vortexed. The suspension was inoculated on roll-tube medium and incubated. Colonies were randomly picked up and inoculated into detection medium that contains 3 × 3 mm filter paper and incubated at 39°C until degradation of the filter paper was observed. One isolate was identified as *C. lentocellum* based on 16S rRNA gene sequence analysis. HNost1007 was anaerobically grown on RF15GC medium at 39°C for 48 h. Genomic DNA was extracted using NucleoBond Buffer Set III and AXG20 (Macherey-Nagel, Düren, Germany), with quality confirmed by the 5200 Fragment Analyzer System and Agilent HS Genomic DNA 50 kb Kit (Agilent Technologies Japan, Ltd., Hachioji, Japan). DNA was fragmented using a g-TUBE (Covaris, Woburn, Massachusetts, USA), and the sequencing library was prepared using the SMRTbell Express Template Prep Kit 2.0 (Pacific Biosciences, Menlo Park, CA, USA) according to the manufacturer’s instructions. Polymerase complexes were generated using the Binding Kit 2.2 (Pacific Biosciences, Menlo Park, CA, USA), and *de novo* sequencing was performed on the PacBio Sequel IIe platform. Adapter sequences were removed using SMRT Link (v.10.1.0.119528) to obtain sub-reads, which were aligned to generate a consensus sequence. HiFi reads were obtained by excluding sequences with an average quality score below 20. Reads shorter than 1,000 bases were removed using Filtlong (ver 0.2.0). High-quality HiFi reads were assembled using Flye (ver 2.9), and the contigs were verified using Bandage (ver 0.8.1). Genome completeness was confirmed using CheckM (ver 1.1.2) (10), and annotation was performed using the DFAST v.1.2.18 pipeline (11). Default parameters were used except where otherwise noted.

The number of raw reads was 21,889. The complete genome of strain HNost1007 consists of a circular 4,639,609 bp chromosome with 98.0% genome completeness and 4.7% contamination values. The genome has a G + C content of 34.4% (Table 1). Final DNA coverage was 46.6. *N*_50_ was 4,639,609. DFAST predicted 4,262 coding sequences (CDSs), 25 rRNA genes, and 103 transfer RNA (tRNA) genes (Table 1). The genome contains three CRISPR arrays.

**TABLE 1:** Genome features of *Cellulosilyticum lentocellum* HNost1007 revealed by DFAST (11)

Attribute	Value
Total sequence length (bp)	4,639,609
Gap ratio (%)	0.0
GC content (%)	34.4
Number of CDSs	4,262
Average protein length (aa)	310.6
Coding ratio (%)	85.6
Number of rRNAs	25
Number of tRNAs	103
Number of CRISPRs	3

## Data Availability

The sequence reads generated in this study have been submitted to the Sequence Read Archive under the accession number DRR701521. The 16S ribosomal RNA gene sequence and the assembled genome have been deposited to DDBJ/ENA/NCBI GenBank under the accession number LC945213 and AP041033, respectively. The BioProject accession number and the BioSample accession number were PRJDB19914 and SAMD00858808, respectively.
